# The TMK Subfamily of Receptor-Like Kinases in Arabidopsis Display an Essential Role in Growth and a Reduced Sensitivity to Auxin

**DOI:** 10.1371/journal.pone.0060990

**Published:** 2013-04-16

**Authors:** Ning Dai, Wuyi Wang, Sara E. Patterson, Anthony B. Bleecker

**Affiliations:** 1 Department of Botany and Laboratory of Genetics, University of Wisconsin-Madison, Madison, Wisconsin, United States of America; 2 Department of Horticulture, University of Wisconsin-Madison, Madison, Wisconsin, United States of America; University of Antwerp, Belgium

## Abstract

Mechanisms that govern the size of plant organs are not well understood but believed to involve both sensing and signaling at the cellular level. We have isolated loss-of-function mutations in the four genes comprising the transmembrane kinase TMK subfamily of receptor-like kinases (RLKs) in Arabidopsis. These TMKs have an extracellular leucine-rich-repeat motif, a single transmembrane region, and a cytoplasmic kinase domain. While single mutants do not display discernable phenotypes, unique double and triple mutant combinations result in a severe reduction in organ size and a substantial retardation in growth. The quadruple mutant displays even greater severity of all phenotypes and is infertile. The kinematic studies of root, hypocotyl, and stamen filament growth reveal that the TMKs specifically control cell expansion. In leaves, TMKs control both cell expansion and cell proliferation. In addition, in the *tmk* double mutants, roots and hypocotyls show reduced sensitivity to applied auxin, lateral root induction and activation of the auxin response reporter DR5: GUS. Thus, taken together with the structural and biochemical evidence, TMKs appear to orchestrate plant growth by regulation of both cell expansion and cell proliferation, and as a component of auxin signaling.

## Introduction

During development, it is essential for all multicellular organisms to perceive and process information from extracellular signals via cell surface receptors to control organ size and growth. In animals, the evolutionarily conserved receptor tyrosine kinase (RTK) signaling pathway plays a central role in regulating cell proliferation, cell growth, cell differentiation and survival [1], [2]. RTKs comprise a large family of integral membrane proteins with highly divergent extracellular domains coupled to a conserved intracellular tyrosine kinase motif. Plant receptor-like kinases (RLK) exhibit a basic structural similarity with animal RTKs, but also display several unique characteristics. Most plant RLKs are serine/threonine kinases and have extracellular domains distinct from ligand-binding motifs of RTKs in animals [3], [4]. Structural features of plant RLKs support the idea that they may also function as entry points for signaling pathways.

In Arabidopsis, there are more than 400 RLKs with divergent extracellular motifs and cytoplasmic serine/threonine kinase domains [3]. While examples are still limited, several plant RLKs have been assigned functions including brassinosteroid signaling (BRI1) [5]–[6], meristem development (CLV1) [7], floral organ abscission (HAESA, HAESA-LIKE2, and EVERSHED) [8]–[10], pollen tube and ovule development (FERONIA) [11], cell wall signaling such as lignin deposition, cell expansion, and cell elongation (FERONIA; THESEUS 1; HERCULES 1 and 2) [12]–[16], drought and salt stress responses [17] and inflorescence development [18]. Ligands have been identified for BRI1 [19], HAESA [9], the systemin and phytosulfokine receptors [20], the ERECTA-family RLKs [21] and the CLV1/BAM family [22]. It has also been shown that closely related RLKs can bind ligands with similar functions and yet that recognition mechanisms are quite complex.

*TMK1* encodes a RLK with leucine-rich repeats in the extracellular domain and was previously found to be expressed in all major organs [23]. This early work also demonstrated autophosphorylation of the kinase domain on serine and threonine residues with a preference for Mn^2+^ and Mg^2+^ [24]. Despite these molecular and biochemical analyses of TMK1, its biological function was not determined. The *TMK1* loss-of-function mutant does not show any growth or developmental abnormality compared to wild type [25], suggesting that other RLKs in Arabidopsis are capable of compensating for the function of TMK1. In this study, we explore the biological function of all four members in the TMK subfamily of RLKs in Arabidopsis and present evidence that the four members of the TMK subfamily play a critical role in the control of cell expansion and cell proliferation. Mutant combinations among the TMKs result in plants that are smaller in size as a consequence of reduced cell size in roots, hypocotyls and stamen filaments, and decreased cell size and cell number in leaves. Hormone assays demonstrated that mutant combinations have reduced sensitivity to auxin in lateral root induction, reduced DR5:GUS activation, and that the *tmk1;tmk3;tmk4* triple mutant is insensitive to applied auxin. Our study indicates that the four members of the TMK subfamily act in a functionally redundant manner to mediate both cell expansion and proliferation downstream of auxin.

## Results

### The TMK Subfamily Members Show Overlapping Patterns of Tissue Expression

**Figure 1:**
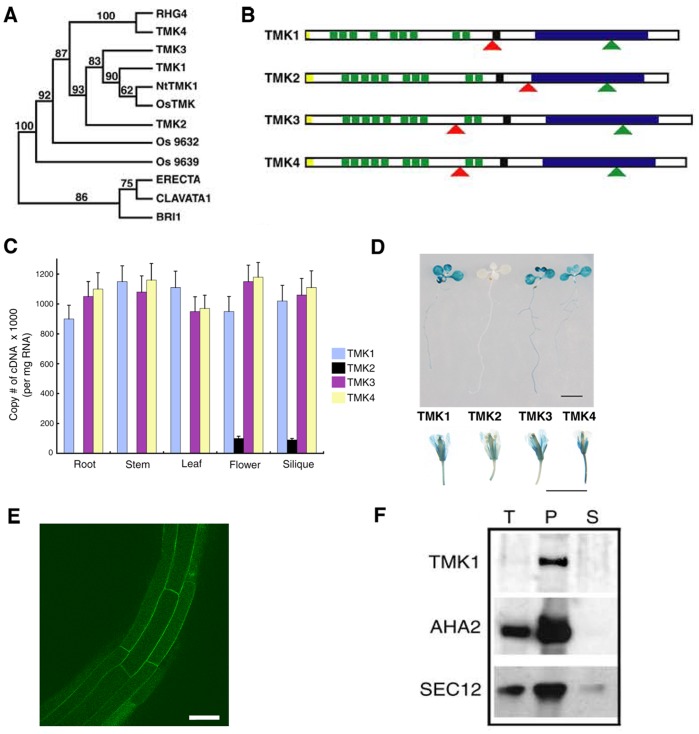
TMK Subfamily of RLKs and Gene Expression Patterns. (A) The phylogenic tree of the TMK subfamily and its closest relatives in flowering plants. TMK1 (At1g66150); TMK2 (At1g24650); TMK3 (At2g01820) and TMK4 (At3g23750) are all from Arabidopsis. OsTMK, Os9632 and Os 9639 are from *Oryza sativa*, NtTMK1 from *Nicotiana tabacum*, and RHG4 from *Glycine max*. (B) Domain organizations showing intron locations (green triangle) and T-DNA positions (red triangle) for each of the TMK family members. Shared features include signal peptide (yellow), multiple leucine rich repeats (green), transmembrane region (black) and kinase domain (blue). (C) Quantitation of transcript abundance for each member of the TMK subfamily of RLKs. TMK1, TMK3 and TMK4 are expressed at approximately equal levels in all organs examined, while TMK2 expression can only be detected in the flowers and siliques. (D) Expression patterns of GUS reporter driven by *TMK1*, *TMK2*, *TMK3* and *TMK4* native promoters in seedlings and flowers. Scale bars: 10 mm. (E) Expression of TMK1 is associated with the plasma membrane in the mature zone of root in Arabidopsis as indicated by TMK1:GFP lines in which the TMK1 promoter was translationally fused with GFP. Scale bar: 50 µm. (F) TMK1 is an integral membrane protein as determined by crude protein extracted from Arabidopsis seedlings. Note expression of other membrane-associated proteins AHA2 and SEC12 as controls. T: total protein, P: membrane pellet, S: soluble fraction.

Phylogenetic analysis of all RLKs from Arabidopsis based on kinase domain sequence alignment revealed a distinct TMK subfamily composed of TMK1 and three other RLKs, designated TMK2, TMK3, and TMK4 (Fig. 1A). Several orthologous sequences are present in rice (*Os*) [26], tobacco (*Nt*) [27] and soybean *RHG4* [28], indicating potential roles for the TMK subfamily in both dicot and monocot species (Fig. 1A). The four members of the Arabidopsis TMK subfamily possess a similar domain organization, containing multiple LRR repeats in the extracellular region with a characteristic intervening sequence separating LRRs into two blocks. In addition, members of the TMK subfamily contain a unique intron in the same location in the kinase domain (Fig. 1B).

To determine expression patterns of the members of the TMK subfamily, a semiquantitative RT-PCR analysis was performed. TMK1, TMK3 and TMK4 were expressed at comparable levels in all organs tested, including roots, stems, leaves, flowers and siliques (Fig. 1C). On the other hand, TMK2 was detected in trace amounts only in flowers and siliques (Fig. 1C). The expression patterns of the TMK subfamily members were further confirmed by analysis of the **β**-glucuronidase (GUS) reporter expressed under the control of their native promoters (Fig. 1D). All four TMKs were expressed in similar tissues in seedlings and flowers; however, TMK 2 expression was significantly lower and only detected when seedlings are exposed in the staining solution for an extended period of time.

### TMK1 Localizes to the Cellular Membranes

Based on the protein sequences, the four members in the TMK subfamily are predicted to be transmembrane proteins. In order to evaluate this prediction, the entire TMK1 coding region was translationally fused with modified green fluorescent protein (GFP) in frame at the C-terminus. This fusion construct TMK1:GFP, driven by the native TMK1 promoter, complements the *tmk1; tmk4* mutant phenotype (Table 1). Confocal microscopic analysis of TMK1:GFP transgenic plants revealed that TMK1 localizes to the cell periphery (Fig. 1E). Western blotting analysis further confirmed TMK1 membrane localization; as shown in Figure 1F in which TMK1 was highly enriched in the pellet fraction containing cellular membranes. TMK1 was not detectable in the soluble fraction.

**Table 1:** Phenotypic characterization of *tmk* mutants.

Genotype	Root Length4DAG (mm)	Etiolated Hypocotyl Length 4DAG (mm)	Maximum Rosette Diameter (cm)	Seeds/Silique
***Wild type***	21.8±1.4	10.6±1.1	7.5±0.9	50.2±7.1
*tmk1*	22.5±1.9	10.2±1.5	7.2±1.0	48.6±6.5
*tmk2*	21.6±1.3	10.0±1.7	6.9±0.7	48.9±9.2
*tmk3*	23.4±1.7	11.2±1.8	8.1±1.1	51.2±6.8
*tmk4*	24.8±1.5	10.5±1.4	7.4±0.8	46.3±8.1
*tmk1;tmk2*	21.2±1.2	10.2±1.7	7.9±0.9	47.2±9.3
*tmk1;tmk3*	22.6±1.5	10.9±1.6	7.1±0.8	42.8±8.7
*tmk1;tmk4*	6.9±0.9*	4.2±0.8*	3.6±0.5*	15.4±3.6*
*tmk2;tmk3*	20.4±1.5	10.7±1.5	7.2±0.7	44.3±7.5
*tmk2;tmk4*	22.1±1.3	10.3±1.7	7.8±0.6	48.6±6.7
*tmk3;tmk4*	21.4±1.4	10.8±1.3	7.5±0.8	45.6±7.8
*tmk1;tmk2;tmk4*	6.6±0.7*	4.4±0.7*	3.1±0.4*	14.6±3.4*
*tmk1;tmk2;tmk3*	21.4±1.1	10.0±0.8	7.6±0.6	49.4±7.1
*tmk1;tmk3;tmk4*	3.4±0.4*	2.6±0.7*	2.1±0.3*	4.6±1.3*
*tmk2;tmk3;tmk4*	19.6±0.8	9.9±1.7	6.9±0.7	50.1±8.3
*tmk1;tmk2;tmk3;tmk4*	3.2±0.3*	2.4±0.6*	2.1±0.4*	0*
*tmk1;tmk4;gTMK1*	21.8±1.7	9.9±1.0	7.6±1.0	42.7±9.1
*tmk1;tmk4;gTMK4*	22.2±1.9	10.2±1.1	7.7±1.1	41.7±11.2
*tmk1;tmk3;tmk4;gTMK3*	6.3±0.6*	4.2±0.8*	3.7±0.9*	11.4±5.4*
*tmk1;tmk2;tmk3;tmk4;gTMK2*	3.4±0.5*	2.8±0.9*	2.3±0.4*	3.8±2.1*
*tmk1;tmk4;gTMK1:GFP*	20.9±1.6	10.4±1.2	7.5±0.9	46.0±6.0

Wild-type plants were compared with various *tmk* mutant combinations and transgenic plants. The average ± standard deviation is shown for each measurement (n>30). Values that are significantly different (p<0.001) from wild type are labeled with an asterisk. 4 DAG: 4 days after germination.

### Combinatorial Loss of Function of *TMK*s in Arabidopsis Markedly Inhibits Growth and Development

**Figure 2:**
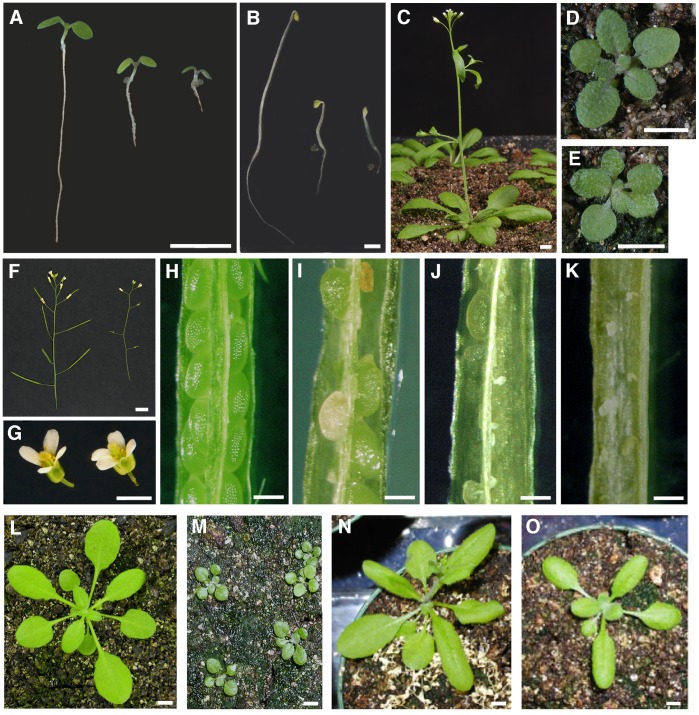
Phenotypic characterization of *TMK* mutants and wild type Arabidopsis show that the *TMK* Genes Act Redundantly to Regulate Diverse Aspects of Plant Growth and Development. (A) Four day-old light grown seedlings (wild type COL; *tmk1; tmk4* double mutant; *tmk1; tmk3; tmk4* triple mutant). (B) Four day-old etiolated seedlings (wild type COL; *tmk1; tmk4* double mutant; *tmk1; tmk3; tmk4* triple mutant). (C) 4 week-old wild-type plant. (D) 4 week-old tm*k1; tmk4* double mutant. (E) 4 week-old *tmk1; tmk3; tmk4* triple mutant. (F) Inflorescence of wild type (left) and quadruple mutant (right). Note sterility. (G) Developing flowers of wild type (left) and quadruple mutant (right). (H) Developing silique of wild type. (I) Developing silique of *tmk1; tmk4* double mutant. (J) Developing silique of *tmk1; tmk3; tmk4* triple mutant. (K) Developing silique of quadruple mutant showing infertility. (L) Rosette of four week old wild type plant. (M) Rosette of four week old *tmk1; tmk4* double mutant plants. (N) Recovery of *tmk1; tmk4* phenotype by gTMK1. (O) Recovery of *tmk1; tmk4* phenotype by gTMK4. Scale bars: 5 mm for (A)–(G) and (L)–(O); 0.5 mm for (H)–(K).

The homozygous *tmk1, tmk2, tmk3* and *tmk4* mutants containing a T-DNA insertion in the coding region of the genes were identified and verified by PCR and sequencing (Fig. 1B). We could not detect the full-length cDNA of any of the corresponding TMKs in the T-DNA insertion lines supporting the selection of homozygous lines for loss of function. All mutants were backcrossed five generations and then carefully observed throughout their entire life cycle, and no significant differences in growth or development relative to wild type were observed in any of the single mutant lines (Table 1). To assess functional redundancy, we constructed all possible double and triple mutants, as well as the quadruple mutant. While most double mutants did not display any significant altered phenotypes (Table 1), the *tmk1; tmk4* double mutant displayed a severe reduction in organ size, a substantial retardation in growth, a delay in development, and a decrease in fertility (Fig. 2; Table 1 and Table 2). The *tmk1; tmk3; tmk4* triple mutant showed increased severity of all phenotypes observed in *tmk1; tmk4* double mutants, while the quadruple mutant was comparable to the triple mutant in vegetative growth (Table 1); however, completely infertile (Fig. 2H and 2K; and Table 1).

**Table 2:** Phenotypic characterization of *tmk* mutants.

Genotype	Days to Bolting	# of Rosette Leaves	Internode Length(cm)	# of Flowers/Primary Inflorescence	Maximum Height/Primary Inflorescence (cm)
Wild type	26.8±3.9	11.2±1.1	0.72±0.18	45.8±4.9	40.8±5.7
*tmk1;tmk4*	42.8±4.6*	11.8±1.3	0.73±0.13	18.9±3.6*	20.6±3.2*
*tmk1;tmk3;tmk4*	49.9±3.8*	10.9±1.4	0.69±0.16	15.7±3.5*	12.1±2.9*
*tmk1;tmk2;tmk3;tmk4*	49.1±4.1*	11.0±1.4	0.70±0.11	11.4±3.1*	9.8±2.1*

Wild-type plants were compared with *tmk* mutants. The average ± standard deviation is shown for each measurement (n>20). Values that are significantly different (p<0.01) from wild type are labeled with an asterisk.

The seedlings of all mutants appeared normal at germination; however, after four days of growth on vertical agar plates, the root length of the *tmk1; tmk4* double mutant was about 1/3 that of wild type and the root length of the *tmk1; tmk3; tmk4* triple mutant was further reduced to approximately 1/6 that of wild type (Fig. 2A and Table 1). Hypocotyls of *tmk1; tmk4* and *tmk1; tmk3; tmk4* etiolated seedlings were three and five times shorter than those of wild type, respectively (Fig. 2B and Table 1). Vegetative shoot development was also retarded as indicated by a chronological delay in flowering time (Table 2 and Fig. 2C–E). Both the *tmk1; tmk4* double and *tmk1; tmk3; tmk4* triple mutants produced the same number of rosette leaves as wild type at bolting, but required a significantly longer time to bolt (Table 2). Rosette diameters of the *tmk1; tmk4* double and the *tmk1; tmk3; tmk4* triple mutants are about half and one third of wild type, respectively (Table 1 and Fig. 2C–E).

In contrast to leaf, hypocotyl and root development, the inflorescence shoot of all mutant combinations was similar to wild type in terms of internode length and flower size (Fig. 2F–G and Table 2). However, the initiation of bolting was delayed, and the height of the primary inflorescence and the length of stamen filaments were significantly reduced (Table 2 and Fig. 2G). Approximately ¼ the number of flowers were produced on the primary inflorescence of the quadruple mutant, and the mutant was completely infertile (Table 2 and Fig. 2K). Reductions in fertility were also observed in the double *tmk1; tmk4* and the triple mutant *tmk1; tmk3; tmk4* (Fig. 2 H–J). In order to further understand the lack of fertility in *tmk* mutant combinations, we tried to restore fertility by pollinating with both mutant and wild-type pollen. We found that pollen from the respective mutant parent lines restored fertility in *tmk1; tmk4* and *tmk1; tmk3; tmk4,* but not the quadruple mutant. Pollination with wild-type pollen also restored fertility in the double and triple mutants yet only partially in the quadruple.

To confirm that the phenotypes of the mutants are caused by the loss of function of *TMK*s, genomic clones of each gene were transformed into mutant backgrounds. Growth of *tmk1; tmk4* was restored to wild type by either the *TMK1* or *TMK4* genomic clone (Table 1 and Fig. 2L–O). Similarly, transformation of *tmk1; tmk3; tmk4* with the *TMK3* genomic clone resulted in partial restoration of the triple mutant phenotype to one similar to, or less severe than, that of the *tmk1; tmk4* double mutant (Table 1). Consistent with the expression pattern of *TMK2*, the genomic clone of *TMK2* partially rescued the infertility of the quadruple mutant (Table 1). As indicated, we obtained complete restoration of fertility with either *TMK1* or *TMK4* alone in the double mutant *tmk1; tmk4*. The partial rescue of *tmk1; tmk3; tmk4* and the quadruple *tmk1; tmk2; tmk3; tmk4* by *TMK2* indicates functional redundancy between all members of the family. In summary, the complete loss of function of *TMK*s in Arabidopsis markedly inhibits growth and development of most organs, including roots, hypocotyls, leaves, and stamen filaments.

### Cellular Defects of *tmk* Mutants

We hypothesized that the reduced organ size in the mutants could be due either to a reduction in cell size, cell number or both. To investigate the cellular basis of the *tmk1; tmk4* double mutant, *tmk1; tmk3; tmk4* triple mutant and quadruple mutant phenotypes, we measured cell size and cell number in roots, hypocotyls, leaves, and stamen filaments.

**Figure 3:**
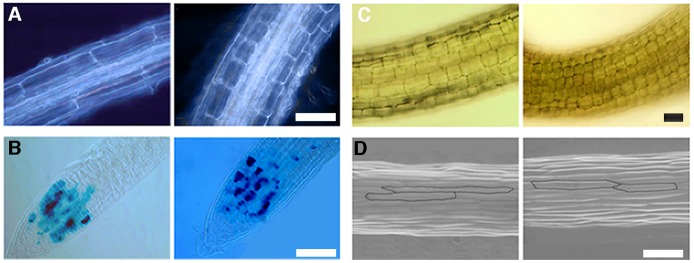
*TMKs* Regulate Cell Expansion in Roots, Hypocotyls and Filaments. (A) Root cortical cells of wild type and *tmk1; tmk4* double mutant showing differences in cell expansion (wild type – left panel; *tmk1; tmk4*- right panel). Scale bar: 100 µm. (B) Expression of the cell division marker *Cyc1At:GUS* in the root meristem of wild type and *tmk1; tmk4* double mutant showing similar patterns of expression (wild type – left panel; *tmk1; tmk4*- right panel). Scale bar: 50 µm. (C) Hypocotyl cortical cells of wild type and *tmk1; tmk4* double mutant showing differences in cell expansion (wild type – left panel; *tmk1; tmk4*- right panel). Scale bar 10 µm. (D) Filament epidermal cells of wild type and *tmk1; tmk4* double mutant showing differences in cell expansion (wild type – left panel; *tmk1; tmk4*- right panel). Scale bar: 50 µm.

In the root, the length of mature cortical cells in the *tmk1; tmk4* double mutants, and *tmk1; tmk3; tmk4* triple mutants is approximately 1/3 and 1/4 that of wild type, respectively (Fig. 3A). However, the number of cortical cells is similar in the mutants and wild type (Table 3). This result was supported by analysis of the cell division marker Cyc1At:GUS [29], which demonstrated that the frequency of cell division in the root meristems of *tmk1; tmk4* was similar to wild type (Fig. 3B). Similar to the defect in roots, the reduction in the length of the *tmk1; tmk4* and *tmk1; tmk3; tmk4* mutant hypocotyls and stamen filaments are most likely due to attenuated cell expansion rather than cell proliferation (Fig. 3C–D and Table 3). These observations clearly suggest that TMKs contribute to the regulation of cell expansion in roots, hypocotyls, and stamen filaments.

**Table 3:** Cellular characterization of roots, hypocotyls and stamen filaments of *tmk* mutants.

Genotype	Root (3 days after germination)			Etiolated Hypocotyl (4 days after germination)			*Stamen Filament* (at anthesis)		
	Length (mm)	Cortical CellLength (µm)	Cortical Cells/File	Length (mm)	Cortical CellLength (mm)	Cortical Cells/File	Length (mm)	Epidermal CellLength (µm)	EpidermalCells/File
Wild type	12.9±1.6	179.2±9.8	48.4±1.5	10.6±1.1	1.6±0.5	22.3±0.8	3.1±0.3	134±24	17.6±2.4
*tmk1;tmk4*	4.3±0.7*	37.6±8.9*	49.1±1.8	4.2±0.8*	0.7±0.2*	22.1±0.7	2.4±0.2*	109±26*	17.2±2.1
*tmk1;tmk3;tmk4*	3.7±0.4*	28.3±6.4*	47.9±2.1	2.6±0.7*	0.6±0.2*	21.7±1.1	1.9±0.2*	93±25*	17.6±2.5
*tmk1;2;3;4*	3.6±0.4*	27.4±7.2*	47.7±2.3	2.4±0.6*	0.5±0.2*	21.9±0.9	1.7±0.1*	89±22*	17.5±2.1

Wild-type plants were compared with *tmk* mutants at the same age (n>20). Cells were measured for each tissue (n>100). The average±standard deviation is shown. Values that are significantly different (p<0.01) from wild type are labeled with an asterisk.

While these observations clearly indicate a role of TMKs in the regulation of cell expansion, we were also interested in determining if rates of cell division were also affected by loss of TMKs. A kinematic analysis of the root growth was performed to gain a deeper understanding of the cellular basis of the short root phenotype of the *tmk1; tmk4* and *tmk1; tmk3; tmk4* mutants. Rates of cell expansion, cell division, and the duration of time that a cell spent in the root elongation zone were calculated [30]. The results show that the effects of the *tmk* mutations on root growth could be attributed to a reduction in both the rate and duration of cell expansion (Table 4).

**Table 4:** Analysis of duration and rate of average root growth in *tmk* mutants.

Genotype	Cell production rate (cells/hour)	Cell residence time (hours)	Average growth rate (1/h)
Wild type	0.97±0.2	25.1±4.1	0.165±0.004
*tmk1;tmk4*	0.94±0.3	14.9±3.6*	0.113±0.008*
*tmk1;tmk3;tmk4*	0.98±0.3	10.4±3.3*	0.119±0.009*

Wild-type plants were compared with *tmk1;tmk4*, and *tmk;tmk3;tmk4* mutants (n = 20). Cell size and cell number from the cortical cell file along different zones of roots were measured for each genotype. Numbers indicate the average ± standard deviation. The average growth rate = [ln(final cell size)−ln(initial cell size)]/residence time. Values that are significantly different (p<0.01) from wild type are labeled with an asterisk.

**Figure 4:**
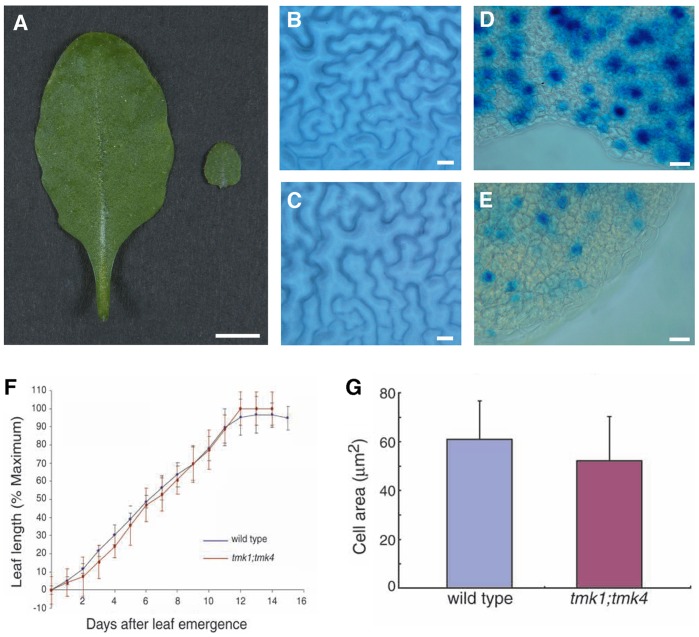
*TMKs* Regulate Cell Proliferation During Leaf Development. (A) Fully expanded leaf 6 of wild type (left) and *tmk1; tmk4* double mutant (right). (B) Cell size of leaf epidermal pavement cells in wild type. (C) Cell size of leaf epidermal pavement cells in the *tmk1; tmk4* double mutant. (D) The frequency of cell division (cells stained with blue/total cells) in the primordia of leaf 6 in wild type. (E) The frequency of cell division (cells stained with blue/total cells) in the primordia of leaf 6 in the *tmk1; tmk4* double mutant. (F) Expansion rate of leaf 6. Leaf emergence was determined, as seedlings emerged with minimum leaf length greater than 2 mm. (G) The size of dividing adaxial epidermal pavement cells (stained with blue) in the primordia of leaf 6 in wild type and in the *tmk1; tmk4* double mutant. Scale bars: (A) = 5 mm; (B)–(C) = 10 µm; (D)–(E) = 20 µm.

In contrast to the finding that the decreased length of *tmk* mutant roots, hypocotyls and stamen filaments is due to a reduction in cell expansion, the decrease in leaf size was primarily due to a reduction in cell number. For example, measurements of mature epidermal pavement cells of *tmk1; tmk4* revealed only a 15% reduction in cell size of the *tmk1; tmk4* mutant versus wild type (Table 5). Since this alone cannot explain the 90% reduction of leaf size in *tmk1; tmk4* (Fig. 4A–C), we hypothesized that cell proliferation is also affected in the mutant leaves. We observed that patterning of cells in the epidermis and overall structure of pavement, guard, and trichome cells were primarily wild type in appearance (Fig. 4B and C). In addition, the leaf expansion rates, measured for the cell expansion phase of leaf development, were indistinguishable between *tmk1; tmk4* and wild type when normalized to the final size (Fig. 4F); thus, suggesting that the mutations primarily affected the early cell proliferation phase of leaf expansion. To visualize the frequency of cell division during early leaf development, we examined Cyc1At:GUS activity in leaf primordia in wild type and the *tmk1; tmk4* double mutant. The frequency of cells with GUS activity in the primordia of *tmk1; tmk4* leaves was only about 1/3 of that in wild-type leaf primordia (Fig. 4D and E, and Table 5). Thus, the decreased leaf size of *tmk1; tmk4* is due in large part to reduced cell proliferation during early leaf development.

**Table 5:** Analysis of cell division and cell size in tmk mutant leaves.

Genotype	Size of the6^th^ leaf (cm^2^)	Size of adaxialmature epidermalpavement cells (µm^2^)	Frequency of cellsshowing Cyc1At:GUSactivity
Wild type	3.04±0.28	1431±412	4.3%±0.6%
*tmk1;tmk4*	0.28±0.08*	1212±376*	1.2%±0.4%*
*tmk1;tmk3;tmk4*	0.15±0.04*	1132±382*	ND
*tmk1;tmk2;tmk3;tmk4*	0.16±0.03*	1118±394*	ND

Wild-type plants were compared with various *tmk* mutant combinations. Adaxial epidermal pavement cells were measured in 20 plants for each genotype. The average±standard deviation is shown. Values that are significantly different (p<0.01) from wild type are labeled with an asterisk. The frequency of cells showing Cyc1At:GUS was determined by number of adaxial epidermal pavement cells stained with Cyc1At:GUS/total number of adaxial epidermal pavement cells. ND: not determined.

In the process of cell division, cell proliferation is often accompanied by cell growth, except in the early stages of embryogenesis in animals [31]. An intracellular mechanism monitors cell growth and halts progression of cell proliferation through the cell cycle at specific G1 or G2 checkpoints if the cell has not grown to a threshold size [32] **.** We hypothesized that the reduced frequency of cell proliferation in the leaf primordia of *tmk1; tmk4* is caused by decreased cell growth as most mutations that affect the cell cycle do not affect organ size [33]. Consequently, we predicted that the size of dividing cells in the leaf primordia of the *tmk1; tmk4* double mutant should be similar to wild type because the *tmk1; tmk4* mutant cells need to reach the critical size for dividing. To test this hypothesis, we measured the size of adaxial epidermal pavement cells that stained positive for Cyc1At:GUS activity. Results revealed that the size of dividing cells of *tmk1; tmk4* double mutants and wild type is similar (Fig. 4G). In conclusion, it is likely that the function of TMKs is to directly regulate cell growth in early proliferative phase of leaf initiation and development, and that leaf cells have a more stringent requirement to reach a certain size before cell division than other cell types.

### Altered Responses of *TMK* Mutants to the Growth Regulator Auxin

**Figure 5:**
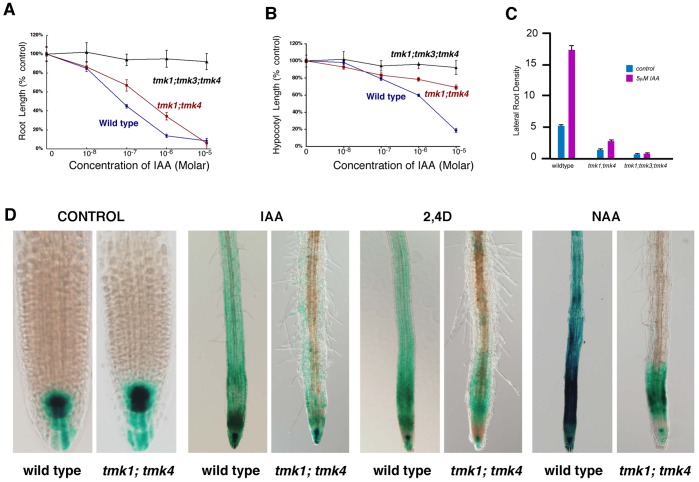
*TMK* mutants display reduced sensitivity to applied auxins. (A) Root length of light grown seedlings: isogenic wild type, *tmk1; tmk4* and *tmk1;tmk3;tmk4.* (B) Hypocotyl length of etiolated seedlings: isogenic wild type, *tmk1; tmk4* and *tmk1;tmk3;tmk4.* (C) Lateral root density of wild type and *tmk1; tmk4*; and *tmk1; tmk3; tmk4* mutants in response to 5.0 µM IAA. (D) Reduced sensitivity of *tmk1; tmk4* mutant to applied auxin as illustrated by expression of DR5:GUS after treatments with 0.1 µM IAA, 2,4-D, and NAA, for 24 hours. Control panel represents no treatment.

Due to our observations on reduction in cell elongation and cell division, we screened *tmk* seedlings for altered responses to growth regulators: abscissic acid (ABA), auxin, cytokinins, ethylene, and gibberellins. Whereas mutant seedlings responded relatively normally to ethylene, ABA and cytokinin (data not shown), the double mutant *tmk1; tmk4* showed reduced sensitivity to applied auxin and the triple mutant *tmk1; tmk3; tmk4* was insensitive to applied IAA (Fig. 5A). Similar results were obtained for etiolated hypocotyls (Fig. 5B). Roots of the mutant also showed a significant reduction in auxin stimulated lateral root development (Fig. 5C). Further evidence for altered auxin sensitivity was obtained by crossing the auxin responsive DR5:GUS reporter gene into the *tmk1; tmk4* background. Whereas the root tip of *tmk1; tmk4* mutant seedlings showed a normal DR5:GUS expression pattern without applied auxin, the elongation zone and part of mature zone of the root showed a greatly reduced response to applied IAA, 2,4-D and NAA (Fig. 5D). This suggests that auxin sensitivity is affected early in the auxin signaling pathway, given that DR5 is a reporter for the activity of AUX/IAA transcriptional factors [34].

## Discussion

We observed that the increasing loss of members of the TMK subfamily of RLKs in Arabidopsis leads to increased severity of a suite of phenotypes without revealing phenotypic effects specific to individual isoforms. The lack of a detectable phenotype in any double or triple mutant combination that leaves a functional TMK1 or TMK4 intact indicates that either of these genes is capable of compensating for the loss of the others. Lastly, the effects of the loss of function of TMK2 are revealed only in the absence of all other isoforms and are restricted to reproductive organs. Taken together, these results indicate that members of the TMK subfamily are largely redundant in function.

The observation that *TMK* genes are required for determining cell numbers without affecting the overall cellular pattern in leaves is particularly intriguing given the receptor-like nature of the TMK proteins and the fact that other members of the RLK family of proteins have been implicated in determining cell numbers in other tissues [18]; [35]–[38]. For example, the CLAVATA1 receptor, along with the cognate ligand CLAVATA3, is responsible for maintaining the population size of cells in the shoot meristem [35]. Another LRR-containing RLK, ERECTA has been implicated in regulating cell numbers in developing inflorescence tissues [36], [39]. Thus CLV1, ERECTA and TMKs all appear to regulate cell proliferation. As ERECTA is most strongly expressed in inflorescence tissue [40], potential functional redundancy between the TMK and ERECTA RLKs could explain why single *tmk* mutants do not show a growth phenotype in the inflorescence despite the fact that they are highly expressed in this tissue. It is also possible that these closely related RLKs can bind similar ligands.

Within a species, organ size exhibits remarkable uniformity under a specific set of environmental conditions. However, the mechanisms for size control are still poorly understood. Current models of organ size determination emphasize either the role of cell proliferation and growth at the cellular level or organ level control systems [41]. The latter viewpoint is supported by recent research in which genetic modifications that alter cell proliferation in developing leaves are compensated partially or completely by changes in cell size, so that the ultimate size of the organ is constant [42], [43]. On the other hand, there are a number of Arabidopsis mutants, particularly hormone-related mutants, with reduced organ size that are affected primarily in cell size [44], [45], while loss of ANT function reduces mature organ size by decreasing cell numbers [46].

The phenotypes of the *tmk1; tmk4* double mutant and *tmk1; tmk3; tmk4* triple mutant leaves are striking in that the size reductions of 10 and 20 fold, respectively, are primarily due to reduction in cell number without any compensation in cell size (Table 5). This effect of *tmk* mutations on cell proliferation in leaves must be reconciled with the primary effect on cell expansion in roots, hypocotyls, and stamen filaments.

Given the fact that post-germination hypocotyl development proceeds almost entirely by cell expansion [47] and seedlings of the *tmk* mutants and wild type are indistinguishable in hypocotyl cell number after germination, it seems clear that *tmk* mutants can directly affect cell expansion in the absence of cell division. Interestingly, antisense suppression of the WAK subfamily of RLKs also leads to a reduction in cell expansion [48]. However, in this case, cell size in all organ systems was affected, including leaves.

It is possible that the *tmk* mutations affect organ size by different mechanisms in leaves versus other tissues or altered responses to hormone regulation. There is precedent for this as the ethylene receptors affect different tissues in different ways [49]. Modulation could be regulated by cellular and tissue specificity as well as temporal. Specific regulatory cofactors yet to be discovered may provide additional information on understanding specificity. A proposed model that we favor is that the loss of TMK activity affects cell growth, and that developing leaves respond differently than roots. This model would predict that cell size may influence cell division. The fact that the cell size of dividing cells of the mutants and wild type is similar in leaves supports this model.

### *tmk* Mutants Showed Reduced Response to Applied Auxin

The growth defects of the *tmk1; tmk4* double and *tmk1; tmk3; tmk4* triple mutants indicate that the TMK genes are required for normal plant growth. While the specific growth phenotypes of these mutants do not fit previously described profiles for mutations in known hormone pathways, it is possible that TMKs are involved in novel aspects of hormone pathways. The insensitivity of the *tmk1; tmk3; tmk4* triple mutant roots and hypocotyls to applied auxin is a stronger phenotype than most existing auxin-resistant mutants, and this reduced sensitivity to applied auxin was also indicated by the suppression of auxin-induced lateral root development and reduction in induction of DR5:GUS expression in the higher order *tmk* mutants. The loss of TMK activity appears to specifically affect auxin signal transduction in root and potentially shoot development. While the role for auxin in cell expansion is well established, understanding auxin’s role in organ development is less well characterized. Further investigation of growth factor(s) and other components involved in the TMKs signal transduction pathway will help us understand plant organ size control at both molecular and cellular levels.

## Methods

### Phylogenetic Analysis

The protein sequence of the TMK1 kinase domain was used to conduct BLAST searches against sequences deposited in the GenBank database. The most closely related proteins with an E value cutoff of 1×10^−90^ and the well-studied Arabidopsis RLKs, CLAVATA1, BRI1 and ERECTA were further used for sequence alignment and phylogenetic analysis. The kinase domain sequences were aligned using MacClade and the phylogenetic tree was generated with PAUP using the neighbor-joining method. The bootstrap values are shown on the nodes [4].

### Cell Fractionation and Immunoblot Analysis

Arabidopsis ecotype Columbia (COL) wild-type seeds were germinated and grown in MS liquid culture. Tissues were harvested after 10 days and homogenized with extraction buffer (20 mM Tris, pH 8.5,150 mM NaCI, 1 mM EDTA, 20% glycerol, and 1 mM PMSF, 1 unit/mL macroglobulin, 1 µg/mL pepstatin, 10 µg/mL aprotinin, and 10 µg/mL leupeptin as protease inhibitors). The homogenate was strained through Miracloth and centrifuged at 100,000 g for 30 min. The pelleted membrane fraction was resuspended in solubilization buffer (10 mM Tris, pH 7.3, 150 mM NaCI, 1 mM EDTA, 10% glycerol, 1% Triton X-100) containing the protease inhibitors listed above, and centrifuged again at 100,000 g for 30 min. Samples were subjected to SDS-PAGE and subsequent Western blot analysis using α-CT antiserum as described [23].

### Growth Conditions

Mutants and wild-type Arabidopsis seeds were surface-sterilized in 10% chlorox for 10 minutes and rinsed with sterile water or 70% (v/v) ethanol for 2 min, dried on sterile filter paper, and placed on 1/2 MSNS (half-strength Murashige and Skoog salts without sucrose) agar plates, or directly sown in soil. Seeds were stratified at 4°C for 2 days in the dark prior to being transferred to a growth chamber (Econair Ecological Chamber, Winnipeg, Canada) set at 22°C in a 16-h light (125 µE m^−2^ s^−1^) and 8-h dark regime. About 7 days after germination, seedlings were transferred to soil, a mixture (2∶1, v/v) of Jiffy Mix (Jiffy products of America, Batavia, IL) and Perlite (Midwest Perlite, Appleton WI), and grown until the plants were photographed or reached maturity.

### Mutant Isolation

The T-DNA insertion lines *tmk3* and *tmk4* for *TMK3* (At2g01820) and *TMK4* (At3g23750), respectively, were isolated following the instructions of the Wisconsin knock-out facility. The *tmk1* mutant for *TMK1* (At1g66150) was identified in the T-DNA Express Collection at the Salk Institute (Salk_016360) [50], and the *tmk2* mutant for *TMK2* (At1g24650) was isolated from the Syngenta collection (Garlic_1242) [51]. All mutant lines were backcrossed five times to COL wild type prior to generation of double, triple, and quadruple mutants for any phenotypic analysis. The isogenic wild type COL was used as control in all phenotypic analyses except when specified as LER. LER lines were generated after selection of mutant lines in COL ecotype and backcrossed five times to LER wild type prior to generation of the double mutant *tmk1; tmk4.* The following gene-specific primers were used for genotyping:

TMK1 (At1g66150), 5′–CGATCCTTGTACTAACTGGATTGGGATA–3′ and 5′–CCGCAACT-GTAATCTTAACACTCTCATT–3′; TMK2 (At1g24650), 5′–CCGAAATCTAGTGGTTCTTCA-TGGTTA–3′ and 5′–GTGTCTCTTCGTT-GACCTCCATTGCTT–3′; TMK3 (At2g01820), 5′–CTGTAGTATTTCGTTGC-GTTCCTACTGAA–3′ and 5′–GAAGAGTGGACCGA-TTCTGCTGATT–3′; and TMK4 (At3g23750), 5′–GACCTAGGAATGTCAGGAACGATCGAA–3′ and 5′–TTCACCGTAGCCGGAAACTTAGGTAT–3′. PCR conditions are: 95°C for 2 m; 95°C 20 s/60°C 20 s/72° 30 s repeated for 34 cycles; 72°C for 10 minutes.

### Semi-quantitative Reverse Transcriptase-mediated Polymerase Chain Reaction (RT-PCR)

Isolation of total RNA, cDNA synthesis, and RT-PCR were carried out as described previously described by Bleecker lab [52], [53]. The TMK1,TMK2, TMK3, and TMK4 PCR products were amplified using gene specific primers, detected by transillumination, photographed with a Kodak digital camera, and quantified with the ImageQuant software (Molecular Dynamics, Sunnyvale, CA). Data analysis was performed as described previously [54]. The following gene-specific primer pairs for each member of the TMK subfamily were used for RT-PCR: TMK1 (At1g66150), 5′-CACCAGAGCTTTATACACAGGGATCTT-3′ and 5′-TCGCTTTCTTGAATGATGCT-3′; TMK2 (At1g24650), 5′-CACAATCAATTGAGACCAAGATT-3′ and 5′ ATCTCGCGGTTCCCTGGAGCTACATTGATT-3′; TMK3 (At2g01820), 5′-AAGGGCGAAAACCGTTAGAC-3′ and 5′-GCATTTTCGTCTTTGCTGGCTGCT-3′, and TMK4 (At3g23750), 5′-ACTCGGTTACTCTCCTTTGACA-3′ and 5′-TGAGAACCACACCAAATGCA-3′.

### Plasmid Construction and Plant Transformation

The genomic fragments of *TMK1* (6055 bp), *TMK2* (5675 bp), *TMK3* (6033 bp), and *TMK4* (5663 bp) were amplified using PCR and cloned into pPZP221 [55] pPZP221B, a derivative of pPZP221 that was generously provided by Sebastian Bednarek (University of Wisconsin, Madison) and in which the *aacC1* gene (gentamycin selectable marker) is replaced with the *bar* gene (glufosinate/bialphos selectable marker) [56]. These genomic clones contain the 5′ flanking region upstream of the start codon and the 3′ flanking region downstream of the stop codon with lengths of 1946 and 1196 bp (*TMK1*), 1977 and 952 bp (*TMK2*), 2011 and 1004 bp (*TMK3*), and 1862 and 869 bp (*TMK4*), respectively. These assignments of nucleotide positions were based on the annotation in the TAIR database.

To generate the β-glucuronidase (GUS) fusion constructs (TMK:GUS), a *Sma* I to *Eco*R I fragment consisting of the GUS gene and the NOS terminator from pBI121 was inserted into pPZP211. The fragments of *TMK1*, *TMK2*, *TMK3*, and *TMK4* consisting of the 5′ flanking regions and the first few N-terminal amino acids were then ligated in frame into pPZP211 upstream of GUS.

For the TMK1:GFP fusion construct, a 370-bp NOS terminator was amplified from the plasmid pCD64 kindly provided by Chris Day (University of Wisconsin, Madison) and inserted into pPZP221B at restriction sites *Pst* I and *Hind* III. An *Eco*R I to *Spe* I *TMK1* fragment consisting of the 5′ flanking region and the complete genomic coding region of the genomic *TMK1* clone and an *Xba* I to *Pst* I EGFP fragment amplified from the plasmid pEGFP were ligated in frame into *Eco*R I and *Pst* I-cleaved pPZP221 such that the NOS terminator was positioned behind EGFP.

All the constructs were confirmed by sequencing and transformed into wild type or mutant Arabidopsis plants using *Agrobacterium tumefaciens* strain ABI and the floral dipping method [57]. T_1_ transgenic plants were selected either on 1/2 MSNS plates containing 50 µg/mL kanamycin for the constructs in pPZP211 and 100 µg/mL gentamycin for the constructs in pPZP221, or on soil by spraying Liberty (glufosinate-ammonium) for the construct in pPZP221B. Homozygous transgenic plants were obtained for each construct based on segregation analysis of resistance to antibiotics or herbicide in the progeny.

### GUS Staining in Plants

The Cyc1At:GUS reporter consists of the Cyc1At promoter plus the region coding for the first 150 amino acids of Cyc1At, including the cyclin destruction box (CDB). The CDB is fused in frame to GUS, so that CDB:GUS protein is degraded at the end of mitosis [29]. To visualize GUS reporter activity, seedlings were incubated in 90% acetone for 1 hour at room temperature and washed three times with GUS staining buffer (100mM NaPO_4_, pH 7) for 5 min each. The wash buffer was then replaced with the GUS staining solution [29] containing 1 mg/ml of 5-bromo-4-chloro-3-indoyl-B-D-glucuronide X-Gluc (Research Organics Cleveland, Ohio) and the samples were incubated 4–6 hours at room temperature in the dark. The reaction was stopped by rinsing with buffer and then replacing the staining buffer with 70% EtOH.

### Microscopic Analysis

Plant materials for light microscopy were examined using a BX60 Olympus microscope with DIC optics (Olympus Optical Company, Tokyo, Japan). Photographic images were recorded using the Olympus DP70 system. For the TMK1:GFP analysis, we used a Bio-Rad MRC-1024 Laser Scanning Confocal Microscope with the 24-bit imaging software (Bio-Rad, Hercules, CA). Developing siliques and stamen filament epidermal cells were imaged with a Quanta 200 Scanning Electron Microscope (FEI Company, Hillsboro, Oregon). The size of epidermal pavement cells was determined from cyanoacrylate glue replicas of the adaxial leaf surface [58]. Measurements were taken from four fields of each leaf, which contained ten or more epidermal cells.

### Root Kinematic Analysis

The Arabidopsis root kinematic analysis was performed as described previously [30]. Seedlings were grown under light conditions for various time intervals, and at specific times, whole mounts of roots were analyzed by microscopy. Cell lengths of all cells in a cortical cell file were plotted against distance from the tip. The root growth rate data and cell file analysis were combined to calculate cell production rate (cells/hour), cell residence time (hours) and average growth rate (1/h):

Cell production rate = root elongation rate (mm/hour)/final cell size (mm/cell).

Cell residence time = Number of cells in elongation zone/cell production rate = hours.

Average growth rate = [ln(final cell size)−ln(initial cell size)]/cell residence time (µm/µm.h [µm. µm–1.–1] or 1/h).

### Lateral Root Analysis

Seeds of *tmk1;tmk4*, *tmk1;tmk3;tmk4* and isogenic wild type were plated and cold treated for three days before placement in the lights. Three days after germination, seedlings were transferred to 1/2 MSNS plates containing different concentrations of hormones and grown vertically for another 4 days. The number of lateral roots was counted and lengths of roots were measured as described previously [59].

### Statistical Analysis

Statistical tests were performed using Mstat 4.01 software (University of Wisconsin, Madison).
